# Clinical and Neuroimaging Features in Charcot–Marie–Tooth Patients with *GNB4* Mutations

**DOI:** 10.3390/life11060494

**Published:** 2021-05-28

**Authors:** Hye Mi Kwon, Hyun Su Kim, Sang Beom Kim, Jae Hong Park, Da Eun Nam, Ah Jin Lee, Soo Hyun Nam, Soohyun Hwang, Ki Wha Chung, Byung-Ok Choi

**Affiliations:** 1 Departments of Neurology, Samsung Medical Center, Sungkyunkwan University School of Medicine, Seoul 06351, Korea; huimei.kwon@samsung.com (H.M.K.); jh0711.park@samsung.com (J.H.P.); 2 Departments of Radiology, Samsung Medical Center, Sungkyunkwan University School of Medicine, Seoul 06351, Korea; hyunsu83.kim@samsung.com; 3 Department of Neurology, Kyung Hee University Hospital at Gangdong, Kyung Hee University College of Medicine, Seoul 06351, Korea; sbkim@khu.ac.kr; 4 Department of Biological Sciences, Kongju National University, Gongju 32588, Korea; daeun5612@naver.com (D.E.N.); jhmom1010@naver.com (A.J.L.); 5 Institute of Stem Cell and Regenerative Medicine, Samsung Medical Center, Seoul 06351, Korea; suhyun.nam@samsung.com; 6 Department of Pathology and Translational Medicine, Samsung Medical Center, Sungkyunkwan University School of Medicine, Seoul 06351, Korea; soohyun.hwang@samsung.com; 7 Department of Health Sciences and Technology, SAIHST, Sungkyunkwan University, Seoul 06351, Korea

**Keywords:** Charcot–Marie–Tooth disease, CMTDIF, *GNB4*, peripheral neuropathy, neuroimaging

## Abstract

Charcot–Marie–Tooth disease (CMT) is the most common inherited peripheral neuropathy. Mutations in the *GNB4* gene cause dominant intermediate CMT type F (CMTDIF). The aim of this study is to investigate phenotypic heterogeneities and characteristics of CMT patients with *GNB4* mutations. We enrolled 1143 Korean CMT families and excluded 344 families with a *PMP22* duplication. We further analyzed the 799 remaining families to find their *GNB4* mutations using whole-exome sequencing (WES). We identified two mutations (p.Gly77Arg and p.Lys89Glu) in three families, among which a heterozygous p.Gly77Arg mutation was novel. In addition, a significant uncertain variant (p.Thr177Asn) was observed in one family. The frequency of the *GNB4* mutation in the Korean population is 0.38% in *PMP22* duplication-negative families. All three families showed *de novo* mutation. Electrophysiological findings regarding the p.Lys89Glu mutation showed that the motor nerve conduction velocity (MNCV) of the median nerve was markedly reduced, indicating demyelinating neuropathy, and sural nerve biopsy revealed severe loss of myelinated axons with onion bulb formation. Lower extremity Magnetic Resonance Imaging (MRI) demonstrated relatively more severe intramuscular fat infiltrations in demyelinating type (p.Lys89Glu mutation) patients compared to intermediate type (p.Gly77Arg mutation) patients. The anterolateral and superficial posterior compartment muscles of the distal calf were preferentially affected in demyelinating type patients. Therefore, it seems that the investigated *GNB4* mutations do cause not only the known intermediate type but also demyelinating-type neuropathy. We first presented three Korean families with *GNB4* mutations and found phenotypic heterogeneities of both intermediate and demyelinating neuropathy. We suggest that those findings are useful for the differential diagnosis of CMT patients with unknown *GNB4* variants.

## 1. Introduction

Charcot–Marie–Tooth disease (CMT) is a genetically and clinically heterogeneous disorder characterized by distal muscle weakness, sensory loss, and areflexia [1,2]. CMT can be classified into three neuropathy types: demyelinating neuropathy with reduced median motor nerve conduction velocity (MNCV, <38 m/s) [3,4], axonal neuropathy with preserved median MNCV (>38 m/s) [5,6], and intermediate neuropathy with MNCV between 25 and 45 m/s [7].

The *GNB4* gene (MIM 610863) encodes guanine-nucleotide-binding protein subunit beta-4 (Gb4), which is widely expressed in many tissues including axons and Schwann cells of peripheral nerves [8,9]. Mutant GNB4 proteins cause problems in the G protein-coupled receptor (GPCR) signaling pathway including dysfunction of nerve cells and axons, peripheral neuropathy, and scoliosis [10,11,12]. Soon et al. suggested a dominant-negative effect of *GNB4* mutant proteins in the GPCR signaling of peripheral nerves as a pathogenic mechanisms [13].

Mutations in the *GNB4* gene cause dominant intermediate CMT type F (CMTDIF, MIM 615185) [13]. Until now, only a small number of CMT families with *GNB4* mutations, including two Han Chinese (p.Gly53Asp and p.Lys89Glu), one Czech (p.Lys57Glu), and one Japanese family (p.Gln220Arg), have been reported [13,14,15]. Based on the nerve conduction study, patients with p.Gly53Asp or p.Gln220Arg mutations showed CMTDIF. However, patients with p.Lys57Glu or p.Lys89Glu exhibited demyelinating neuropathy with very slow median MNCVs (12 and 20 m/s, respectively). Therefore, we suggest that the examined *GNB4* mutations had phenotypic heterogeneity including intermediate neuropathy and demyelinating neuropathy.

Lower leg MRI is a useful method for quantifying neuromuscular disease such as CMT [16]. MRI is useful not only as a diagnostic tool but also as a biomarker in clinical trials for CMT therapeutics [17]. It is said that “CMT patients can show different fatty infiltration patterns between demyelinating CMT1A and axonal CMT2A patients”. However, phenotypic heterogeneity and lower leg MRI in CMT patients with *GNB4* mutations have not yet been reported. Here, we present three Korean CMT patients with *GNB4* mutations and analyze their clinical, electrophysiological, and neuroimaging characteristics.

## 2. Patients and Methods

### 2.1. Patients

A cohort of 1,889 CMT patients from 1143 unrelated Korean families from April 2005 to March 2020 was enrolled in this study. We excluded 344 families with a *PMP22* duplication and further analyzed the 799 remaining families to find their *GNB4* mutations using whole-exome sequencing (WES). Written informed consent was obtained from all participants in accordance with a protocol approved by the Institutional Review Boards of Sungkyunkwan University, Samsung Medical Center (2014-08-057-002) and Kongju National University (KNU-IRB-2018-62).

### 2.2. Molecular Genetic Studies

Genomic DNA was extracted from whole blood samples using the QIAamp DNA Mini Kit (Qiagen, Hilden, Germany). Whole exome sequencing was applied on the affected individuals negative for *PMP22* duplication. Exomes were captured using the SureSelect Human All Exon 50M Kit (Agilent Technologies, Santa Clara, CA, USA), and subsequent sequencing was performed using the HiSeq 2500 Genome Analyzer (Illumina, San Diego, CA, USA).

The human genome assembly hg19 was used as a reference sequence (http://genome.ucsc.edu, assessed on 15 July 2016. From the functionally significant variants (missense, nonsense, exonic indel, and splicing site variants), unreported or rare variants with allele frequencies of ≤0.01 were selected in the CMT-related genes. The allele frequencies of the selected variants were checked against the dbSNP (http://www.ncbi.nlm.nih.gov, assessed on 20 January 2021), the 1000 Genomes Project database (1000G, http://www.1000genomes.org/, assessed on 20 January 2021), the Exome Variant Server (EVS, http://evs.gs.washington.edu/EVS/, assessed on 20 January 2021), the Exome Aggregation Consortium Browser (ExAC, http://exac.broadinstitute.org/, assessed on 20 January 2021), and the Korean Reference Genome Database (KRGDB, http://coda.nih.go.kr/coda/KRGDB/, assessed on 20 January 2021). Their exact sequences were confirmed by Sanger sequencing using the genetic analyzers ABI3130XL or by SeqStudio (Life Technologies-Thermo Fisher Scientific, Foster City, CA, USA).

Conservation analysis of the mutation sites was performed using MEGA6, ver. 6.0 (http://www.megasoftware.net/, assessed on 23 January 2021). In silico analysis was performed using the following software: PROVEAN (http://provean.jcvi.org/seq_submit.php, assessed on 23 January 2021), PolyPhen-2 (http://genetics.bwh.harvard.edu/pph2/, assessed on 23 January 2021), and MUpro (http://www.ics.uci.edu/~baldig/mutation, assessed on 23 January 2021). For protein structure prediction and modeling, I-TASSER (https://zhanglab.ccmb.med.umich.edu/I-TASSER, assessed on 25 January 2021) was used [18], and these 3D structures were visualized using the Mol* feature of the Protein Data Bank (http://www.rcsb.org, assessed on 29 January 2021) [19]. In essence, pathogenicity was determined according to the American College of Medical Genetics and Genomics (ACMG) guidelines [20].

### 2.3. Clinical Assessment

Clinical information was obtained in a standardized manner and included assessment of motor and sensory impairments, deep tendon reflexes, and muscle atrophy. The strength of flexor and extensor muscles was assessed manually using the standard Medical Research Council (MRC) scale [21]. In order to determine physical disability, we used two scales, a functional disability scale (FDS) [22] and the CMT neuropathy score version 2 (CMTNS v2) [23]. Sensory impairments were assessed in terms of the perceptual level and severity of pain, temperature, vibration, and position. The age at disease onset was determined by asking patients when and at what age symptoms, i.e., distal muscle weakness, foot deformity, or sensory change, first appeared.

### 2.4. Electrophysiological Examination

Motor and sensory conduction velocities of the median, ulnar, radial, peroneal, tibial, and sural nerves were determined by standard methods using surface stimulation and recording electrodes. MNCVs and compound muscle action potentials (CMAPs) of the median, ulnar, and radial nerves were determined by stimulating at the elbow and wrist. In the same way, the MNCVs and CMAPs of peroneal and tibial nerves were determined by stimulating at the knee and ankle. CMAP amplitudes were measured from baseline to negative peak values. Sensory nerve conduction velocities (SNCVs) were obtained over a finger–wrist segment from the median, ulnar, and radial nerves by orthodromic scoring and were also recorded for sural nerves. Sensory nerve action potential (SNAP) amplitudes were measured from positive peaks to negative peaks.

### 2.5. Sural Nerve Biopsy

A sural nerve biopsy was performed in female patient FC 780 at the age of 17 years. One sural nerve fragment was fixed in 10% formalin, embedded in paraffin, and stained with hematoxylin–eosin (H–E). Another fragment was immediately fixed by immersion in 5% buffered glutaraldehyde and post-fixed in 1% osmium tetroxide. Epon-embedded semi-thin and ultra-thin sections were prepared for light and ultra-structural examinations. Biopsy fragments were examined by electron microscopy.

### 2.6. Lower Extremity MRI

Lower extremity axial MRI of the pelvic girdle, bilateral thigh, and lower leg was reviewed. MRI scans were obtained from 3 *GNB4* patients in a supine position using a 3.0-T MRI system (Skyra, Siemens Healthcare, Frankfurt, Germany). The axial T1-weighted turbo spin-echo images of the thigh and lower leg muscles were graded by the fatty infiltration based on a five-point semiquantitative scale described by Goutallier et al. [24]. T1-weighted spin-echo images were chosen for analysis as this imaging sequence is considered to be optimal for evaluating chronic muscle denervation [25,26]. T1-weighted spin-echo image demonstrates increased signal intensity resulting from fatty infiltration, even with the potential counterbalancing effect of extracellular water content on T1 relaxation time [27]. Three levels (proximal, mid, and distal) were evaluated in thigh muscles, and two levels (proximal and distal) were bilaterally analyzed in the lower leg muscles.

## 3. Results

### 3.1. Identification of GNB4 Mutations

**Figure 1:**
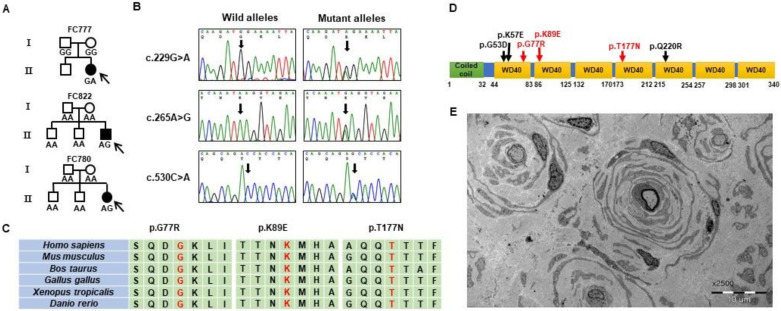
Identification of *de novo GNB4* mutations in Korean patients with CMT. (**A**) Three pedigrees having CMT patients with *GNB4* mutations: FC777 CMTDIF family with c.G229A and FC780 and FC822 CMT1 families both with c.A265G. Genotypes of the corresponding mutations are indicated at the bottom of the plot for all the examined individuals. Black and white symbols represent affected and unaffected individuals, respectively. The probands are indicated by an arrow (□: male, and ○: female). (**B**) Sequencing chromatograms of the *GNB4* mutations. The mutation sites of c.G229A, c.A265G, and c.C530A are indicated by an arrow. (**C**) Conservation of three missense mutation sites. The amino acid sequences in the mutation sites and surrounding regions are very well conserved among vertebrate species. (**D**) Schematic domain structure of GNB4 protein. All the (likely) pathogenic mutations are located in the Trp-Asp repeat (WD40) domains. (**E**) Electron micrograph, sural nerve. The cross-section shows severe loss of large myelinated fibers with frequent onion bulb formations of Schwann cells around hypomyelinated or demyelinated axons.

We identified two (likely) pathogenic variants and an uncertain significant variant from *GNB4* in four CMT families (Supplementary Table S1). A heterozygous *GNB4* (NM_021629.4) c.G229A (p.Gly77Arg) mutation was found in an affected woman with intermediate CMT (family ID: FC777), while her unaffected parents did not have the same mutation, suggesting *de novo* mutation (Figure 1a). This mutation was not reported in the public 1000G, ExAC, EVS, and KRGDB databases. In silico analysis of PRO and Polyphen-2 suggested pathogenic prediction. This mutation was classified as “likely pathogenic (LP)” by the ACMG guideline. A GNB4 (NM_021629.4) c.A265G (p.Lys89Glu) mutation was identified in two demyelinating CMT patients (family ID: FC780 and FC822). In both patients, their unaffected parents and brothers had no same mutation, suggesting *de novo* mutation (Figure 1a). This mutation was reported in a Han Chinese family with CMTDIF (Figure 1d) [5]. A c.C530A (p.Thr177Asn) mutation was found in an isolated patient with mild CMT symptoms of uncertain type (family ID: FC787). This mutation was evaluated as “variant of uncertain significant (VUS)” by the ACMG guideline, and in silico analysis predicted a non-pathogenic variant. The patient’s CMT type was uncertain, and in silico analysis predicted a non-pathogenic classification. This mutation was not reported in the 1000G, ExAC, or EVS; however, it was reported in the KRGDB with a very low allele frequency (0.0006). Therefore, it may be a rare, Korean-specific polymorphic allele.

**Figure 2:**
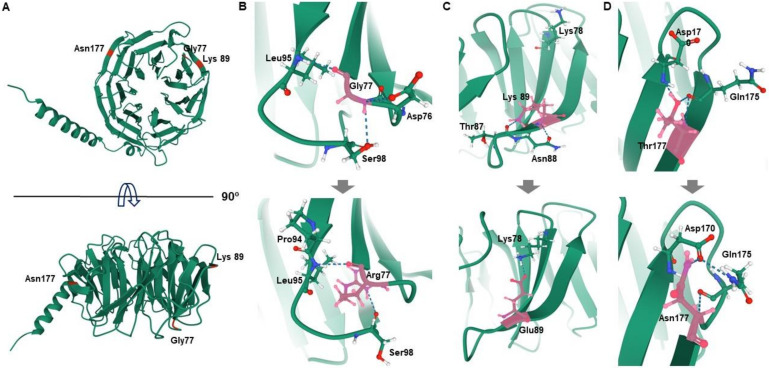
Prediction of 3D structures surrounding the mutation regions in the GNB4 protein. The conformational changes caused by the mutations were visualized using the Mol* feature of the Protein Data Bank. Wild type Gly77, Lys89, and Thr177 residues (top) and their mutant amino acids (bottom) are indicated by the pink colour. Hydrogen bonds are indicated by dotted lines, and hydrogen, carbon, nitrogen, and oxygen atoms are represented in white, green, blue, and red, respectively. (**A**) Ribbon diagrams of the whole *GNB4* gene. The mutation sites are indicated by red. (**B**) p.Gly77Arg. (**C**) p.Lys89Glu. (**D**) p.Thr177Asn.

As a result of predicting the protein 3D structure using the I-TASSER, the C-score was 1.22 in both p.Gly77Arg and p.Lys89Glu mutations, and 1.18 in the p.Thr177Asn mutation. In the p.Gly77Arg mutant, changed from polar to basic, hydrogen bonds with surrounding amino acids were altered as the size of the Arg residue increased, and the hydrogen bond distance between Leu95 and Pro94 was increased. In addition, hydrogen bonds with Asp76 were broken. In the p.Lys89Glu mutation, where a basic amino acid was substituted to an acidic residue, hydrogen bonds with Thr87 and Asn88 were broken, and a bond with p.lys78 located on the upper adjacent domain sheet was newly formed, causing structural modification. In the p.Thr177Asn mutation, where substitution was between amino acids with the same polar property, hydrogen bonds with Asp170 and Gln175 were maintained, but their corresponding atoms and distances were changed (Figure 2).

### 3.2. Clinical Manifestations

The clinical features of the three patients are shown in Table 1. The onset age of patients with the p.Lys89Glu mutation was younger (before 10 years) than that of patients with the p.Gly77Arg mutation (in the third decade of life). Clinical findings confirmed that patients with the p.Lys89Glu mutation were more severely affected than patients with the p.Gly77Arg mutation. In p.Lys89Glu mutation, muscle weakness and atrophy started and predominated in the distal portions of the leg and were noted to a lesser extent distally in the upper limbs. However, in the patient with the p.Gly77Arg mutation, muscle atrophy was not found, and muscle weakness was similar in extent in the upper and lower limbs. Vibratory sensation was reduced to a greater extent than the perception of pain in all three patients. In patients with the p.Gly77Arg mutation, pinprick was normal in the lower limbs. Deep tendon reflexes were absent in patients with the p.Lys89Glu mutation but were decreased in patients with the p.Gly77Arg mutation. Foot deformities were found in all patients. However, scoliosis was found only in patients with the p.Lys89Glu mutation, i.e., not in the p.Gly77Arg mutation (Supplementary Figure S1). Pyramidal signs were not found, and Romberg signs were abnormal in all patients. Functional disability was severe in patients with p.Lys89Glu (FDS = 3 or 4), but a patient with p.Gly77Arg showed low FDS (FDS = 1). The CMTNS v2 of p.Lys89Glu patients was categorized as moderate to severe (CMTNS v2 = 18 and 26); however, the p.Gly77Arg patient was in the mild category (CMTNS v2 = 9).

### 3.3. Electrophysiological Findings

Motor and sensory conduction studies were carried out on three patients (Table 2). The MNCV of our patients with p.Lys89Glu ranged from 3.9 to 7.1 m/s, and that of patients with the p.Gly77Arg mutation ranged from 37.5 to 39.7 m/s. Electrophysiological findings also confirmed that patients with the p.Lys89Glu mutation were more severely affected than those with the p.Gly77Arg mutation. Not all of the sensory nerve action potentials were evoked in p.Lys89Glu patients; however, SNAPs were relatively preserved in p.Gly77Arg patients. We carried out follow-up nerve conduction studies over a 5-year period in a patient with the p.Gly77Arg mutation and observed slow disease progression.

### 3.4. Histopathological Findings

Sural nerve biopsy was performed in a patient (FC 780, II-3) with p.Lys89Glu. The patient showed severe loss of myelinated axons, with onion bulb formation of Schwann cells around hypomyelinated or demyelinated axons (Figure 1e).

### 3.5. Lower Limb MRI Features

**Figure 3:**
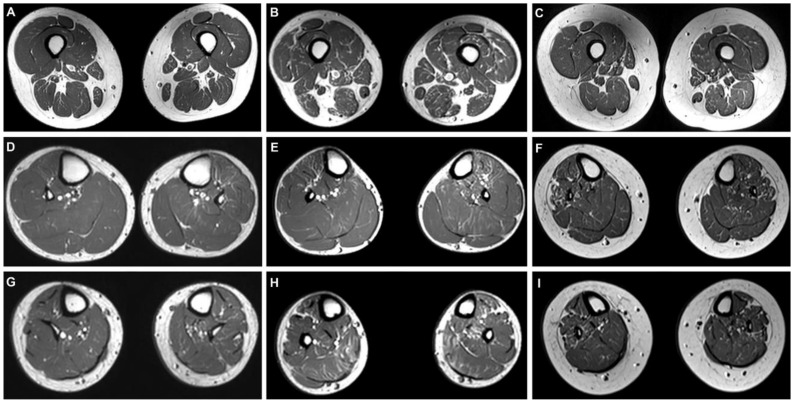
Axial T1-weighted MRIs of the distal thigh (top), proximal (middle) and distal calf (bottom) in FC777 II-2, FC822 II-3, and FC780 II-3. FC777 II-2 (female, 32 years old) shows only minimal fat infiltration in thigh and calf muscles (**A**,**D**,**G**). Both FC822 II-3 (male, 17 years old) and FC780 II-3 (female, 20 years old) show mild fat infiltration in the anterior compartment muscles of the distal thigh (**B**,**C**). FC822 II-3 shows moderate fat infiltration in the soleus muscle at the distal calf and mild fat infiltration in the anterior compartment of the proximal and distal calf (**E**,**H**). FC780 II-3 shows mild fat infiltration in lateral compartment muscles of the proximal and distal calf (**F**,**I**).

Lower limb MRI findings are summarized in Supplementary Tables S1 and S2. Lower limb MRIs were taken twice with an interval of three or five years for each patient. MRIs were obtained at the ages of 27 and 32 for FC777 (II-2), while MRIs for p.Lys89Glu mutation patients were taken at a relatively younger age: FC822 (II-3), 14 and 19; FC780 (II-3), 15 and 18. FC777 (II-2) showed only minimal fat infiltration in calf and thigh muscles without notable progression between follow-up intervals. FC822 (II-3) showed moderate (grade 3) fat infiltration in the soleus muscle at the distal calf and mild fat infiltration in anterior compartment muscles of the distal calf. FC780 (II-3) showed mild fat infiltration in the lateral compartment muscles of the distal calf. FC822 (II-3) showed mild fat infiltration in the anterior and posterior compartment muscles of the distal thigh. FC780 (II-3) also showed mild fat infiltration in the anterior compartment muscles of the distal thigh, demonstrated by a slight progression of fat infiltration in the vastus lateralis and vastus medialis muscles. FC822 (II-3) also showed mild fat infiltration in the posterior compartment muscles of the distal thigh. Otherwise, no significant progression of fat infiltration was noted (Figure 3).

## 4. Discussion

We described the first known Korean families with *GNB4* mutations, including a novel p.Gly77Arg mutation. The p.Lys89Glu mutation in two independent demyelinating CMT patients was previously reported in a Han Chinese family [13], and the p.Gly77Arg mutation in the dominant intermediate CMT patient had not been reported in global public genome databases or the Korean genome database (KRGDB). The frequency of the *GNB4* mutation in the Korean population is determined to be 0.38% in *PMP22* duplication-negative CMT families. The mutation sites are highly conserved among vertebrate animals, and in silico analyses suggested pathogenic prediction of the mutations (Figure 1b,c) [28]. Furthermore, prediction of the *GNB4* protein’s 3D structure suggested conformational changes in mutant proteins (Figure 2). We also identified an additional rare variant, p.Thr177Asn, in a CMT patient of uncertain type with mild symptoms. This mutation was reported in the Korean genome database and was predicted to be non-pathogenic by in silico analyses. We determined this variant as “VUS” according to the ACMG guideline.

The family carrying the same p.Gly53Asp mutation as the Han Chinese family exhibited clinical variability from mild to severe. Patients with mild phenotypes presented nearly normal NCS values, and patients with severe disease phenotypes exhibited demyelinating sensorimotor polyneuropathy with axonal loss [13]. Moreover, the *GNB4* mutation has been described as causing CMTDIF since it was reported as a dominant intermediate neuropathy. However, our patient with the p.Lys89Glu mutation definitely classified into demyelinating neuropathy. Electrophysiological findings of p.Lys89Glu patients showed that the MNCV of median nerves were markedly reduced, ranging from 3.9 m/s to 7.1 m/s, and SNAPs were not recordable in all tested nerves. We observed severe loss of large myelinated fibers and frequent onion bulb formations in the sural nerve biopsy of patient FC780, II-3 (Figure 1e). In addition, the p.Lys89Glu patient reported by Soong et al. showed slow MNCV (20 m/sec), and the Czech patient also showed very slow MNCV (12 m/sec) [13,14]. Conclusively, we suggest that those four unrelated CMT patients with GNB4 mutations could be classified with demyelinating neuropathy. Guanine-nucleotide-binding proteins are heterotrimeric, composed of α, β, and γ subunits, and Gβ4 is abundant in Schwann cells and axons in peripheral nerves [9,10]. This might explain why the *GNB4* mutation could cause demyelinating neuropathy or axonopathy. Therefore, the *GNB4* mutations cause not only the known intermediate neuropathy but also demyelinating neuropathy.

Interestingly, we found that the *GNB4* mutations in all the three examined families arose by *de novo* mutations. Two *de novo* mutations in *GNB4* have been previously reported in Han Chinese and Czech patients [13,14]. So far, seven families with *GNB4* mutations have been reported, and five of them have *de novo* mutations. Therefore, the frequency of *de novo* mutation was high (83.3%). In the case of p.Lys89Glu mutation, the high frequency of *de novo* mutation may be due to the low probability of giving birth due to severe clinical symptoms including scoliosis. This phenomenon may be an accidental discovery, but further research is needed with more patients. In the p.Gly53Asp family, Soong et al. found that men tended to be more severely affected than women [13]. We also found a similar phenomenon. In the two patients with the same mutation (p.Lys89Glu), the male (FC822, II-3) had an earlier age of onset and a more severely affected phenotype than the female (FC780, II-3).

Lower limb MRI findings of the three *GNB4* patients showed generally mild fat infiltrations into lower extremity musculature. MRIs obtained within 3–5 years after diagnosis showed insignificant changes in terms of muscular fat infiltration. The most notable finding demonstrated through MRI analyses was a relatively more severe degree of intramuscular fat infiltration in p.Lys89Glu patients when compared to that of p.Gly77Arg patients. The difference could be deemed more striking considering the age of patients with the p.Gly77Arg mutation is much older than that of p.Lys89Glu mutation patients. The MRI findings are also in agreement with clinical manifestations, showing that p.Lys89Glu mutation patients are more severely affected. Anterolateral and superficial posterior compartment muscles of the distal calf seem to the preferentially affected muscular structures in p.Lys89Glu mutation patients.

There were several limitations to our study. First, population data for race-specific *GNB4* mutations are limited. Second, only three *GNB4* patients (two patients with the p.Lys89Glu mutation and one patient with the p.Gly77Arg mutation) were examined. Therefore, this study has a limitation on the number of patients. However, CMT patients with the *GNB4* mutation are extremely rare, and so far only nine have been reported worldwide.

In summary, we described the first three Korean families with a *GNB4* mutation and found phenotype heterogeneities among intermediate and demyelinating neuropathy. Additionally, we presented lower limb MRI findings of patients with *GNB4* mutations for the first time. These results would be useful for differential diagnosis of CMT patients with unknown *GNB4* variants and for broadening our knowledge of the spectrum of genotypic/phenotypic correlations.

## Figures and Tables

**Table 1:** Clinical characteristics in Charcot–Marie–Tooth patients with *GNB4* mutations.

Patient ID	FC777 (II-2)	FC822 (II-3)	FC780 (II-3)
Mutation ^a^			
Nucleotide	c.G229A	c.A265G	c.A265G
Amino acid	p.Gly77Arg	p.Lys89Glu	p.Lys89Glu
Sex	Female	Male	Female
Age at onset (years)	25	9	5
Age at exam (years)	31	12	17
Disease duration (years)	6	3	12
Muscle weakness ^b^			
Upper limb	+	++	++
Lower limb	+	+++	+++
Muscle atrophy	No	Moderate	Mild to moderate
Pinprick sensation ^c^			
Upper limb	Mildly reduced	Reduced	Reduced
Lower limb	Normal	Reduced	Reduced
Vibratory sensation ^c^			
Upper limb	Reduced	Reduced	Reduced
Lower limb	Reduced	Reduced	Reduced
DTR (knee jerk)	Decreased	Absent	Absent
Scoliosis	No	Yes	Yes
Romberg sign	Abnormal	Abnormal	Abnormal
Pyramidal sign	No	No	No
Foot deformities	Yes	Yes	Yes
FDS ^d^	1	4	3
CMTNS v2	9	26	18
Sural nerve biopsy	ND	ND	Demyelinating neuropathy, onion bulb formation

CMTNS v2 = CMT neuropathy score version 2; DTR = deep tendon reflex; FDS = functional disability scale; ND = not done. ^a^ RefSeq ID for *GNB4*: NM_021629.4 (nucleotide) and NP_067642.1 (amino acid); ^b^ Muscle weakness in upper limbs: + represents intrinsic hand weakness of 4/5 on the MRC scale; ++ represents intrinsic hand weakness < 4/5 on the MRC scale. Muscle weakness in lower limbs: + represents ankle dorsiflexion of 4/5 on the Medical Research Council (MRC) scale; ++ represents ankle dorsiflexion < 4/5, Muscle weakness; +++ represents proximal weakness; ^c^ Pinprick and vibration: mildly reduced represents < 50% sensory loss; reduced represents ≥ 50% sensory loss; ^d^ FDS: 0 = normal; 1 = normal but with cramps and fatigability; 2 = an inability to run; 3 = walking difficulty but still possible while unaided; 4 = walking with a cane; 5 = walking with crutches; 6 = walking with a walker; 7 = wheelchair-bound; 8 = bedridden.

**Table 2:** Electrophysiological features in Charcot–Marie–Tooth patients with *GNB4* mutations.

Patient ID	FC777 (II-2)				FC822 (II-3)			FC780 (II-3)		
Mutation	p.Gly77Arg				p.Lys89Glu			p.Lys89Glu		
Site	Right	Left	Right	Left	Right		Left	Right		Left
Age at examination (years)	26	26	31	31	12		12	17		17
Motor nerve conduction study										
Median nerve										
DTL (ms)	4.2	4.1	4.1	4.0	16.4	25.0		14.2	9.4	
CMAP (mV)	14.9	16.0	13.6	15.4	1.4	0.7		1.5	1.9	
MNCV (m/s)	38.5	37.5	39.7	38.4	4.8	3.9		6.3	7.1	
Ulnar nerve										
DTL (ms)	4.3	3.5	3.4	4.2	12.8	19.0		10.7	13.1	
CMAP (mV)	9.8	12.0	11.4	12.0	1.8	0.5		1.5	1.0	
MNCV (m/s)	33.9	36.8	36.8	34.4	4.4	4.5		4.1	5.5	
Radial nerve										
DTL (ms)	4.0	4.3	4.2	4.4	A	A		A	A	
CMAP (mV)	6.2	5.1	6.3	4.2	A	A		A	A	
MNCV (m/s)	46.4	54.2	48.1	48.1	A	A		A	A	
Peroneal nerve										
DTL (ms)	6.9	6.3	6.4	6.3	A	A		A	A	
CMAP (mV)	2.7	2.9	2.6	2.2	A	A		A	A	
MNCV (m/s)	22.3	24.7	23.2	26.5	A	A		A	A	
Tibial nerve										
DTL (ms)	5.9	5.6	6.1	5.5	A	A		A	A	
CMAP (mV)	10.5	9.9	6.2	4.6	A	A		A	A	
MNCV (m/s)	26.3	26.8	25.9	26.5	A	A		A	A	
Sensory nerve conduction study										
Median nerve										
SNAP (μV)	21.0	20.5	17.2	143	A	A		A	A	
SNCV (m/s)	31.2	33.0	32.8	33.2	A	A		A	A	
Ulnar nerve										
SNAP (μV)	12.4	15.9	12.7	10.9	A	A		A	A	
SNCV (m/s)	28.7	30.3	30.3	32.2	A	A		A	A	
Radial nerve										
SNAP (μV)	13.4	15.7	14.8	14.2	A	A		A	A	
SNCV (m/s)	30.4	31.8	31.8	33.3	A	A		A	A	
Sural nerve										
SNAP (μV)	6.5	5.2	6.8	4.9	A	A		A	A	
SNCV (m/s)	26.4	28.0	28.0	31.1	A	A		A	A	

A = absent action potential; CMAP = compound muscle action potential; SNAP = sensory nerve action potential; SNCV = sensory nerve conduction velocity. The units for the amplitudes of evoked responses are given in parentheses. Normal DTL values: motor median nerve, <3.6 ms; ulnar nerve, <2.5 ms; radial nerve, <3.1 ms; peroneal nerve, <4.8 ms; tibial nerve, <5.1 ms. Normal NCV values: motor median nerve, ≥50.5 m/s; ulnar nerve, ≥51.1 m/s; radial nerve, <57.2 m/s; peroneal nerve, ≥41.2 m/s; tibial nerve, ≥41.1 m/s; sensory median nerve, ≥39.3 m/s; ulnar nerve, ≥37.5 m/s; radial nerve, <44.3 m/s; sural nerve, ≥32.1 m/s. Normal amplitude values: motor median nerve, ≥6 mV; ulnar nerve, ≥8 mV; radial nerve, ≥7 mV; peroneal nerve, ≥1.6 mV; tibial nerve, ≥6 mV; sensory median nerve, ≥8.8 μV; ulnar nerve, ≥7.9 μV; radial nerve, ≥10.0 μV; sural nerve, ≥6.0 μV.

## Data Availability

All raw genetic and clinical data generated or analyzed during this study are available upon request to the corresponding author.

## Supplementary Materials

The following are available online at https://www.mdpi.com/article/10.3390/life11060494/s1, Table S1: Three *GNB4* mutations in the Korean CMT patients, Table S2: Thigh MRI features in Charcot–Marie–Tooth patients with *GNB4* mutations, Table S3: Calf MRI features in Charcot–Marie–Tooth patients with *GNB4* mutations, Figure S1: Whole spine radiograph of FC822, II-3.

## Abbreviations

GPCR	G protein-coupled receptor
C-score	Confidence score for estimating the quality of predicted models by I-TASSER
CMTNS v2	CMT neuropathy score version 2
DTR	deep tendon reflex
FDS	functional disability scale
ND	not done
MRC scale	Medical Research Council scale
CMAP	compound muscle action potential
SNAP	sensory nerve action potential
SNCV	sensory nerve conduction velocity
