# Supplementary material for: Clinical and Neuroimaging Features in Charcot–Marie–Tooth Patients with GNB4 Mutations
Source: Life (Basel). 2021 May 28;11(6):494. doi: 10.3390/life11060494 (PMC8227704; doi:10.3390/life11060494)
Supplement: Supplementary file 1 [file life-11-00494-s001.zip › life-1209544-supplementary.pdf]

Table S1. Three *GNB4* mutations in the Korean CMT patients.

Family ID	Phenotype	Mutation		dbSNP accession No.	Mutant Allele Frequency				*In silico* Analysis			Note	Reference
		Nucleotide	Amino acid		1000G	ExAC	EVS	KRGDB	PROVEAN	PolyPhen-2	MUpro		
FC777	CMTDIF	c.G229A	p.G77R	rs1553851490	UR	UR	UR	UR	-7.064*	0.999*	0.2804	LP	This study
FC822<br>FC780	CMT1<br>CMT1	c.A265G	p.K89E	rs387907341	UR	UR	UR	UR	-3.662*	0.996*	-0.375*	P	Soong et al. 2013
FC787	Uncertain	c.C530A	p.T177N	rs1274879698	UR	UR	UR	0.0006	-2.337	0.084	0.1339	VUS	This study

* Pathogenic prediction; 1000G = 1000 Genomes database; ExAC = Exome Aggregation Consortium Browser; EVS = Exome Variant Server; KRGDB = Korean Reference Genome Database; LP = likely pathogenic; P = pathogenic; UR = unreported; VUS = variant of uncertain significance.

Table S2. Thigh MRI features in Charcot-Marie-Tooth patients with *GNB4* mutations.

Patient	Sex	Age at MRI	Level	Anterior Compartment					Medial Compartment				Posterior Compartment		
				Sartorius	Rectus Femoris	Vastus Intermedius	Vastus Lateralis	Vastus Medialis	Adductor Longus	Adductor Brevis	Adductor Magnus	Gracilis	Semitendinosus	Semimembranosus	Biceps Femoris
FC777	F	27	Proximal	1/1	0/0	1/1	1/1	1/1	1/1	1/1	1/1	1/1	1/1	N/A	N/A
			Mid	1/1	1/1	1/1	1/1	1/1	1/1	1/1	1/1	1/1	1/1	1/1	1/1
			Distal	1/1	0/0	1/1	1/1	1/1	N/A	N/A	N/A	1/1	1/1	1/1	1/1
		32	Proximal	1/1	0/0	1/1	1/1	1/1	1/1	1/1	1/1	1/1	1/1	N/A	N/A
			Mid	1/1	1/1	1/1	1/1	1/1	1/1	1/1	1/1	1/1	1/1	1/1	1/1
			Distal	1/1	0/0	1/1	1/1	1/1	N/A	N/A	N/A	1/1	1/1	1/1	1/1
FC822	M	12	Proximal	1/1	1/1	1/1	1/1	1/1	1/1	1/1	1/1	1/1	1/1	N/A	N/A
			Mid	1/1	1/1	1/1	1/1	1/1	1/1	1/1	1/1	2/2	1/2	1/1	2/2
			Distal	1/1	1/0	1/2	2/2	1/1	N/A	N/A	N/A	1/1	2/2	2/2	2/2
		17	Proximal	1/1	1/1	1/1	1/1	1/1	1/1	1/1	1/1	1/1	1/1	N/A	N/A
			Mid	1/1	1/1	1/1	1/1	1/1	1/1	1/1	1/1	2/2	1/2	1/1	2/2
			Distal	1/1	1/0	1/2	2/2	1/1	N/A	N/A	N/A	1/1	2/2	2/2	2/2
FC780	F	14	Proximal	1/1	1/1	1/1	1/1	1/1	1/1	1/1	1/1	1/1	1/1	N/A	N/A
			Mid	1/1	1/1	1/1	1/1	1/1	1/1	1/1	1/1	1/1	1/1	1/1	1/1
			Distal	1/2	1/1	1/1	1/1	1/1	N/A	N/A	N/A	1/1	1/1	1/1	1/1
		17	Proximal	1/1	1/1	1/1	1/1	1/1	1/1	1/1	1/1	1/1	1/1	N/A	N/A
			Mid	1/1	1/1	1/1	1/1	1/1	1/1	1/1	1/1	1/1	1/1	1/1	1/1
			Distal	1/2	1/1	1/1	1/2*	1/2*	N/A	N/A	N/A	1/1	1/1	1/1	1/1

Right/left side. The presence of fatty infiltration based on a five-point semiquantitative scale described by Goutallier et al. : grade 0, normal; grade 1, some fatty streaks; grade 2, less fat than muscle; grade 3, fatty degeneration of 50%; and grade 4, fatty in filtration of more than 50%

Asterisk indicates an interval increase in the Goutallier grade compared to previous MRI findings

Table S3. Calf MRI features in Charcot-Marie-Tooth patients with *GNB4* mutations.

Patient	Sex	Age at MRI	Level	Anterior compartment			Lateral compartment	Superficial posterior compartment				Deep posterior compartment		
				Tibialis anterior	Extensor digitorum longus	Extensor hallucis longus	Peroneus longus	Gastrocnemius medial	Gastrocnemius lateral	Soleus medial	Soleus lateral	Tibialis posterior	Flexor digitorum longus	Flexor hallucis longus
FC777	F	27	Proximal	1/1	1/1	1/1	1/1	1/1	0/0	1/1	1/0	0/0	N/A	N/A
			Distal	0/0	1/1	1/1	1/1	N/A	N/A	1/1	0/0	0/1	0/1	0/1
		32	Proximal	1/1	1/1	1/1	1/1	1/1	0/0	1/1	1/0	0/0	N/A	N/A
			Distal	0/0	1/1	1/1	1/1	N/A	N/A	1/1	0/0	0/1	0/1	0/1
FC822	M	12	Proximal	2/2	1/2	N/A	1/1	1/1	1/1	1/1	1/1	1/1	N/A	N/A
			Distal	2/2	1/1	1/1	2/1	N/A	N/A	3/3	3/3	1/2	1/1	1/1
		17	Proximal	2/2	1/2	N/A	1/1	1/1	1/1	1/1	1/1	1/1	N/A	N/A
			Distal	2/2	1/1	1/1	2/1	N/A	N/A	3/3	3/3	1/2	1/1	1/1
FC780	F	14	Proximal	1/1	1/1	N/A	2/2	1/1	1/1	1/1	1/1	1/1	N/A	N/A
			Distal	1/1	1/1	1/1	1/2	N/A	N/A	1/1	1/1	1/1	1/1	1/1
	F	17	Proximal	1/1	1/1	N/A	2/2	1/1	1/1	1/1	1/1	1/1	N/A	N/A
			Distal	1/1	1/1	1/1	1/2	N/A	N/A	1/1	1/1	1/1	1/1	1/1

Right/left side. The presence of fatty infiltration based on a five-point semiquantitative scale described by Goutallier et al. : grade 0, normal; grade 1, some fatty streaks; grade 2, less fat than muscle; grade 3, fatty degeneration of 50%; grade 4, fatty in filtration of more than 50%.

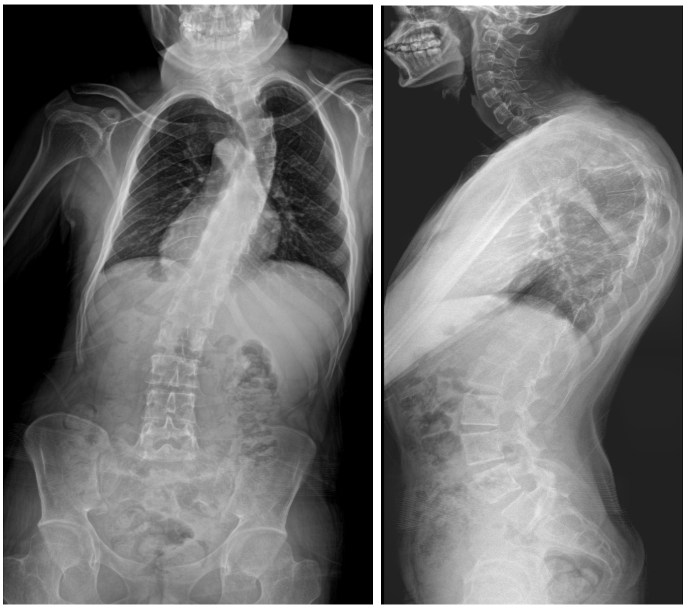

Figure S1. Whole spine radiograph of FC822, II-3; Whole spine PA and lateral radiograph shows prominent kyphoscoliosis in the thoracic spine with accentuated lumbar lordosis.
